# Circulating microRNAs are associated with Pulmonary Hypertension and Development of Chronic Lung Disease in Congenital Diaphragmatic Hernia

**DOI:** 10.1038/s41598-018-29153-8

**Published:** 2018-07-16

**Authors:** Marisol Herrera-Rivero, Rong Zhang, Stefanie Heilmann-Heimbach, Andreas Mueller, Soyhan Bagci, Till Dresbach, Lukas Schröder, Stefan Holdenrieder, Heiko M. Reutter, Florian Kipfmueller

**Affiliations:** 1 Division of Genomics, Life & Brain Research Centre and Institute of Human Genetics, University Hospital Bonn, Sigmund-Freud-Str. 25, 53127 Bonn, Germany; 2 Department of Neonatology and Pediatric Intensive Care, Centre for Pediatrics, University Hospital Bonn, Adenauerallee 119, 53113 Bonn, Germany; 3 Institute for Clinical Biochemistry and Clinical Pharmacology, University Hospital Bonn, Sigmund-Freud-Str. 25, 53127 Bonn, Germany

## Abstract

Pulmonary hypertension (PH) contributes to high mortality in congenital diaphragmatic hernia (CDH). A better understanding of the regulatory mechanisms underlying the pathology in CDH might allow the identification of prognostic biomarkers and potential therapeutic targets. We report the results from an expression profiling of circulating microRNAs (miRNAs) in direct post-pulmonary blood flow of 18 CDH newborns. Seven miRNAs differentially expressed in children that either died or developed chronic lung disease (CLD) up to 28 days after birth, compared to those who survived without developing CLD during this period, were identified. Target gene and pathway analyses indicate that these miRNAs functions include regulation of the cell cycle, inflammation and morphogenesis, by targeting molecules responsive to growth factors, cytokines and cellular stressors. Furthermore, we identified hub molecules by constructing a protein-protein interaction network of shared targets, and ranked the relative importance of the identified miRNAs. Our results suggest that dysregulations in miRNAs let-7b-5p, -7c-5p, miR-1307-3p, -185-3p, -8084, -331-3p and -210-3p may be detrimental for the development and function of the lungs and pulmonary vasculature, compromise cardiac function and contribute to the development of CLD in CDH. Further investigation of the biomarker and therapeutic potential of these circulating miRNAs is encouraged.

## Introduction

Congenital diaphragmatic hernia (CDH) is a rare developmental defect of the diaphragm, causing considerable morbidity and mortality^1,2^. Morphological changes are present from an early developmental stage, consisting of increased muscularization of the pulmonary vessels, rarefication of pulmonary arterioles and capillaries, and a decreased alveolar density^3^. Consecutively, most CDH newborns present with pulmonary hypertension (PH) and lung hypoplasia which are associated with mortality^4^. The severity of PH and lung hypoplasia may subsequently contribute through several mechanisms, such as mechanical ventilation-induced lung damage, to long-term oxygen dependency and the development of chronic lung disease (CLD), which can be defined as oxygen dependency at 28 days of life. The cellular and molecular mechanisms initiating and contributing to PH and CLD are only partially understood^5^. However, morphological changes are likely being enhanced in response to hypoxia, inflammation, oxidative stress and injury of the bronchoalveolar system and the pulmonary vessels^6^.

Therefore, the development of CLD can be considered to be, in a large proportion, induced by the morphological changes leading to PH and lung hypoplasia, and mechanical ventilation-induced lung damage^7,8^. The introduction of lung protective ventilation strategies (e.g. permissive hypercapnia) has improved the outcome of CDH newborns and supportive techniques such as extracorporeal membrane oxygenation (ECMO) may limit lung trauma and improve cardiopulmonary dysfunction^9,10^.

The understanding of the underlying mechanisms for the development of CLD in CDH might lead to the identification of novel biomarkers and drug targets to therapeutically promote lung and vascular growth, which may improve outcome of CDH^11^. MicroRNAs (miRNAs), a class of non-coding RNAs that suppress gene translation (most commonly through promoting mRNA degradation or disrupting mRNA translation), might serve as biomarker for disease severity in CDH^5^. Animal and cellular models of PH suggest that dysregulations of certain miRNAs contribute to the pathogenesis of PH, which might be a predecessor of the development of CLD in CDH^5^. Moreover, miR-200b and miR-10a have been identified to play a crucial role in hypoplastic CDH lungs, and to be associated with survival after fetoscopic endoluminal tracheal occlusion (FETO)^12^. In the present preliminary study, we sought to identify circulating miRNAs as a potential biomarker associated with severe outcomes in CDH newborns, and to uncover specific contributor genes and biological pathways that may aid our understanding of the condition.

## Results

### Cohort characterization

About 72% of patients in our study survived to discharge. ECMO rate was 55.6% (10/18). In 16/18 patients (88.9%), the diagnosis was established prenatally, and 13/18 patients had a left-sided hernia. Eight patients (44.4%) were allocated to the Death/CLD group, while 10 (55.6%) to the No-CLD group. Patients were similar according to sex, gestational age, birth weight and umbilical artery pH. Although patients in the Death/CLD group had a lower O/E lung-to-head ratio (LHR), a higher proportion of intrathoracic liver herniation and presented more right-sided hernias, these differences did not reach statistical significance. However, outcome data were significantly worse in infants in the Death/CLD group, in terms of overall survival, use of ECMO, surgical/patch repair, and duration of mechanical ventilation and oxygen supplementation (Table 1). The individual clinical condition, including the cause of death, of the 18 CDH newborns at the time of blood sample acquisition is demonstrated in Supplementary Table 1.

**Table 1:** Sample demographics and clinical characteristics.

	Death/CLD	No-CLD	p-value
	(n = 8)	(n = 10)	
***Demographics***			
Male, n (%)	4 (50%)	8 (80%)	0.315
Gestational age, weeks (range)	37.6 (34.3–38.4)	38.2 (35.1–39.9)	0.274
Birth weight, kg (range)	3.0 (2.5–3.9)	3.1 (2.2–4.2)	0.897
Umbilical artery pH, median (range)	7.30 (7.25–7.41)	7.35 (7.27–7.45)	0.315
O/E LHR, % (range)	32 (29–46)	42 (35–55)	0.094
Left-sided CDH, n (%)	4 (50%)	9 (90%)	0.213
Intrathoracic liver, n (%)	7 (87.5%)	4 (40%)	0.101
FETO, n (%)	1 (13%)	1 (10%)	0.911
***Time of Sampling***			
Age at blood sampling, hours (range)	24.9 (23.3–29.9)	24.1 (23.4–27.9)	0.315
PaO2 at time of sampling, Torr (IQR)	75 (45–83)	173 (72–193)	0.021
Mild PH, n (%)	0	5 (50%)	0.084
Moderate PH, n (%)	4 (50%)	3 (30%)	0.503
Severe PH, n (%)	4 (50%)	2 (20%)	0.308
***Outcome/Therapies***			
Alive at 28 days, n (%)	5 (63%)	10 (100%)	0.203
Overall survival, n (%)	3 (38%)	10 (100%)	0.027
ECMO support, n (%)	8 (100%)	2 (20%)	0.003
Duration ECMO support, days (range)	12.1 (5.2–34.6)	4.7 (3.1–6.3)	0.267
Surgical Repair, DOL (range)	11 (7–36)	5 (2–11)	0.001
Patch Repair, n (%)	7/7 (100%)	4 (40%)	0.043
Mechanical ventilation, days (range)	36.4 (11.1–53.6)	7.8 (5.3–11.84)	0.001
Oxygen supplementation, days (range)	49.2 (16–262.5)	13.4 (5.8–25.9)	0.005

CLD: chronic lung disease, O/E: observed vs. expected, LHR: lung-to-head ratio, FETO: fetoscopic endoluminal tracheal occlusion, IQR: interquartile range, PH: pulmonary hypertension, ECMO: extracorporeal membrane oxygenation, DOL: day of life.

### Circulating miRNAs associated with severe outcomes in CDH

**Figure 1:**
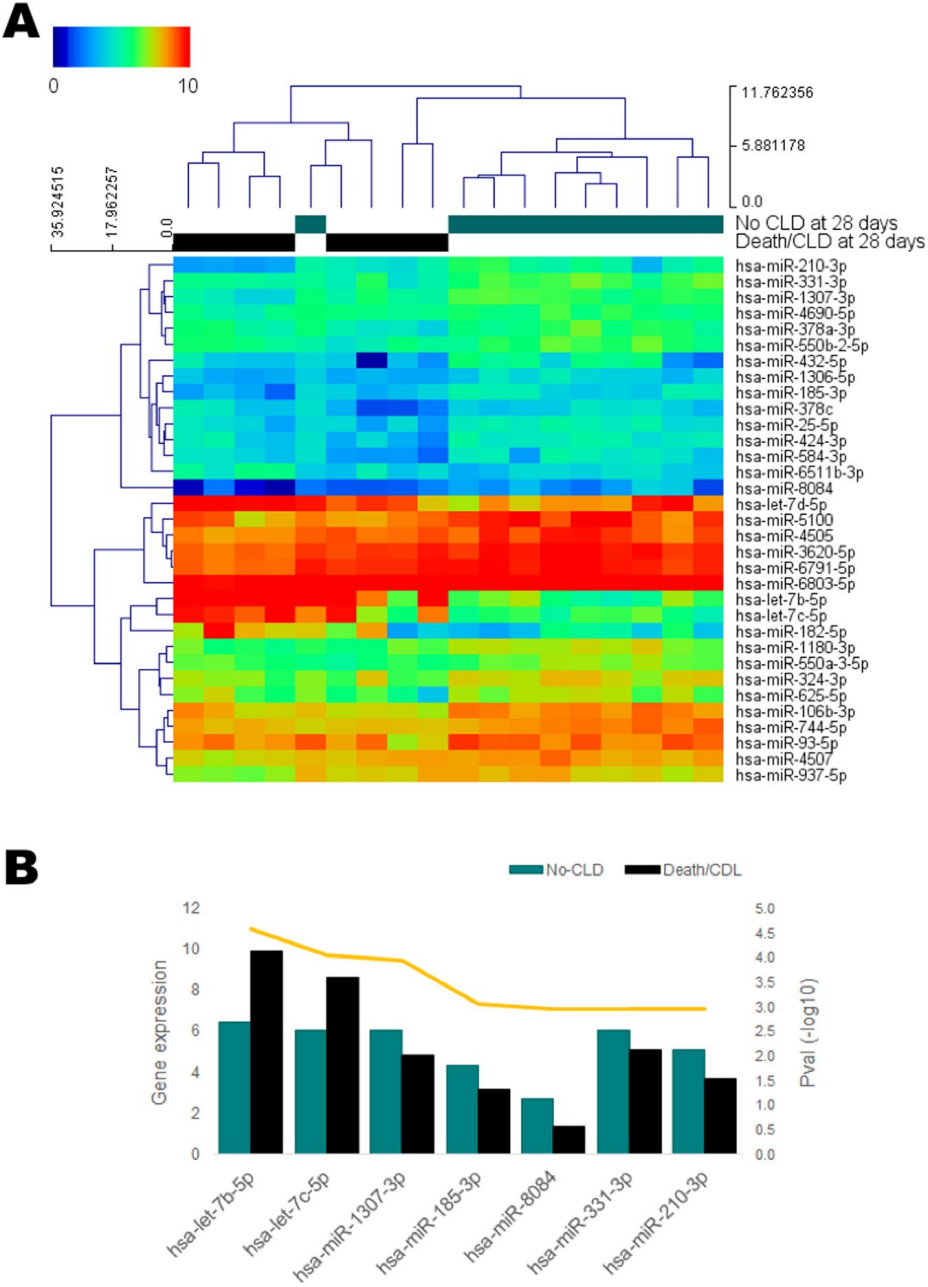
(**A**) Heatmap of significantly changed circulating mature miRNAs in CDH-PH children who died or developed CLD during the first 28 days after birth (Death/CLD at 28 days, n = 8), compared to those children who survived without developing CLD during this period (No-CLD at 28 days, n = 10). Blood samples were drawn 24 h after birth. Statistical significance was obtained through a moderated t-test (unadjusted p < 0.05). Hierarchical clustering was applied to samples and miRNAs using Euclidean distances with complete linkage. **(B)** Expression levels of those differentially expressed miRNAs (Benjamini-Hochberg-adjusted p < 0.05 and expression change ≥1) in the Death/CLD and No-CLD groups of CDH children. P-values are represented by the yellow line.

Through array profiling of mature miRNAs in blood of CDH newborns, collected 24 h after birth, we identified 33 circulating miRNAs that were significantly changed (p < 0.05) in those children who died or developed CLD within 28 days (Death/CLD group) (Fig. 1A, Supplementary Table 2), compared to those who survived with no signs of CLD until up to 28 days of life (No-CLD group). Considering the reduced size of our sample, we used a combined endpoint of death and CLD as a representation of severe disease outcomes. Seven of the 33 significant miRNAs survived correction for multiple comparisons (BH-adjusted p < 0.05, Table 2, Fig. 1B) with an expression change of at least 1, and were thus considered differentially expressed (DE). We observed down-regulation of most of the significant miRNAs in the Death/CLD group. Most miRNAs also appear to have, in average, a reported medium expression in the lung, with considerable numbers of target mRNAs and proteins in this organ, according to the IMOTA database.

**Table 2:** Differentially expressed miRNAs in blood of CDH children with poor outcome at 28 days after birth.

Transcript ID/miRNA	Mean	Mean Death/CLD	Change	*Diff*. *expression*		*Lung-specific information (IMOTA)*		
	No-CLD			Pval	Adj Pval	Expression	mRNAs	Proteins
hsa-let-7b-5p	6.42	9.89	3.47	2.59E-05	0.009	High	1960	463
hsa-let-7c-5p	6.07	8.66	2.60	8.83E-05	0.014	High	1381	271
hsa-miR-1307-3p	6.04	4.82	−1.22	1.14E-04	0.014	Medium	191	42
hsa-miR-185-3p	4.30	3.15	−1.15	8.90E-04	0.046	Not expressed	62	11
hsa-miR-8084	2.71	1.39	−1.32	1.08E-03	0.046	Not found in database		
hsa-miR-331-3p	6.09	5.09	−1.00	1.09E-03	0.046	Medium	617	154
hsa-miR-210-3p	5.12	3.68	−1.44	1.14E-03	0.046	Medium	129	27

*Only miRNAs with Adj Pval <0.05 from a moderated t-test and expression change ≥1 were considered differentially expressed. Blood samples were drawn at a 24 h after birth time-point. CLD: chronic lung disease. Death refers to intra-hospital death between 24 h and 28 days after birth.

**Figure 2:**
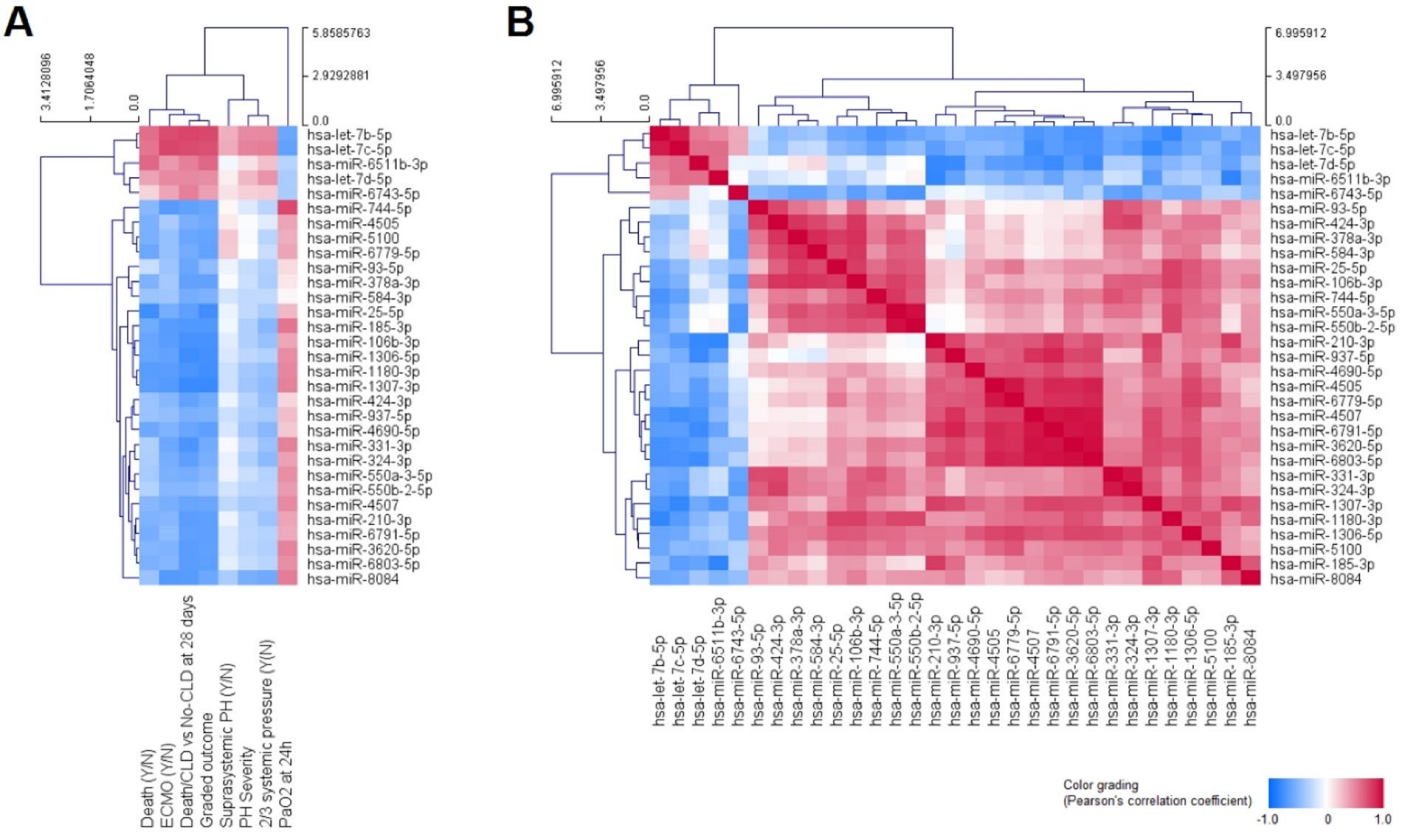
Correlation heatmaps of expression levels of significantly changed miRNAs with clinical classifications/measures **(A)** and the expression of other miRNAs **(B)**. Color grading corresponds to the positive (red) and negative (blue) Pearson’s correlation coefficients. Rows and columns were hierarchically clustered by Euclidean distance with complete linkage. Clinical classifications/measures were mostly represented as absent (N = 0) or present (Y = 1), except for the graded outcome (where no-CLD = 0, CLD = 1 and death = 3), PH severity (where mild = 0, moderate = 1 and severe = 3), and the continuous values of PaO_2_ at 24 h.

Interestingly, miR-6511b-3p (r = 0.621) and miR-25-5p (r = −0.77) showed a relatively high correlation with death, although the number of death cases in our study is too low to consider this a truly significant finding, warranting further exploration. Additional potentially interesting relationships include the negative correlation of miR-8084 expression with moderate to severe PH (r = −0.549), using an established classification of PH severity in this population, and the positive correlation of miR-744-5p levels with PaO_2_ measures at 24 h (r = 0.741) (Fig. 2A). As expected, most significant miRNAs correlated with the outcome, represented as either the binomial No-CLD or Death/CLD classification, or as a graded No-CLD → CLD → Death classification. Furthermore, some DE miRNAs mildly correlated as well with the use of ECMO support and PaO_2_, including let-7b-5p (r_ECMO_ = 0.727, r_PaO2_ = −0.601), let-7c-5p (r_ECMO_ = 0.755, r_PaO2_ = −0.64) and miR-8084 (r_ECMO_ = −0.693, r_PaO2_ = 0.537). However, it is difficult to tell whether these relationships could be the result of ECMO-induced changes already present by the time of sample collection, or could truly result from the abnormal biological processes that ultimately determined the need for ECMO support.

Four major clusters of significant miRNAs can be appreciated from the correlation patterns amongst their expression levels, with the seven DE miRNAs distributed in three of these clusters (Fig. 2B), which might relate to differential functions between clusters and/or to different sources of the circulating miRNAs.

### Functional implications of miRNA dysregulation in the development of CLD in CDH children

We desired to use the miRSystem online tool to investigate target genes of our DE miRNAs, because of the advantages provided by its integrative analysis. However, only four of the seven DE miRNAs (let-7b-5p, let-7c-5p, miR-210-3p and miR-331-3p) were present in this system, for which the target genes for the remaining DE miRNAs (miR-185-3p, miR-1307-3p and miR-8084) were obtained from independent queries in miRTargetLink. Our integrated list, consisting of 857 genes with validated status or a minimum of three hits from miRSystem, and genes from miRTargetLink with some degree of evidence, resulted in the identification of 411 genes predicted to be targeted by more than one of our seven DE miRNAs (Supplementary Table 3). No common target for all DE miRNAs was identified; the largest hits were observed for *SEMA4G*, which was predicted to be targeted by four DE miRNAs, while the highest observed/expected (O/E) ratios, based on the miRSystem integrative analysis, were observed for *NRTN* and *ESPL1*, targeted by the let-7 family of DE miRNAs (Table 3).

**Table 3:** Top target genes for the seven differentially expressed miRNAs in the Death/CLD group of CDH children.

Gene	Hits	miRNAs	Pathways*	O/E*
SEMA4G	4	let-7b-5p, let-7c-5p, miR-210–3p, miR-331–3p	1	2.34
SOCS1	3	let-7b-5p, let-7c-5p, miR-331–3p	26	8.45
UNC5A	3	let-7b-5p, let-7c-5p, miR-331–3p	9	3.57
TBKBP1	3	let-7b-5p, let-7c-5p, miR-331–3p	1	3.37
HMGA1	3	let-7b-5p, let-7c-5p, miR-185–3p	13	3.11
IGF2BP2	3	let-7b-5p, let-7c-5p, miR-331–3p	2	2.79
KCNC4	3	let-7b-5p, let-7c-5p, miR-331–3p	3	2.77
SLC6A1	3	let-7b-5p, let-7c-5p, miR-210–3p	9	2.43
POU2F2	3	let-7b-5p, let-7c-5p, miR-210–3p	2	2.36
EGR3	3	let-7b-5p, let-7c-5p, miR-331–3p	2	1.85
VAV3	3	let-7b-5p, let-7c-5p, miR-185–3p	30	1.64
ACSL6	3	let-7b-5p, let-7c-5p, miR-1307–3p	10	1.56
THBS1	3	let-7b-5p, let-7c-5p, miR-8084	18	1.54
TGFBR1	3	let-7b-5p, let-7c-5p, miR-331–3p	23	1.51
MEF2D	3	let-7b-5p, let-7c-5p, miR-331–3p	12	1.45
ADCY9	3	let-7b-5p, let-7c-5p, miR-185–3p	59	1.44
RAB5B	3	let-7b-5p, miR-331–3p, miR-185–3p	4	1.40
ABCC5	3	let-7b-5p, let-7c-5p, miR-185–3p	3	1.09
NRTN	2	let-7b-5p, let-7c-5p	4	23.67
ESPL1	2	let-7b-5p, let-7c-5p	2	22.19
GNG5	2	let-7b-5p, let-7c-5p	48	16.90
ERCC6	2	let-7b-5p, let-7c-5p	11	16.90
QARS	2	let-7b-5p, let-7c-5p	6	16.90
AKR1B10	2	let-7b-5p, let-7c-5p	4	16.90
HTR1E	2	let-7b-5p, let-7c-5p	9	13.65
NGF	2	let-7b-5p, let-7c-5p	40	11.45
IL8	2	let-7b-5p, let-7c-5p	45	3.29
RANBP2	2	let-7b-5p, let-7c-5p	43	3.20
TP53	2	let-7b-5p, let-7c-5p	64	2.31
NRAS	2	let-7b-5p, let-7c-5p	93	2.25
CHUK	2	let-7b-5p, let-7c-5p	109	2.23
PAK1	2	let-7b-5p, let-7c-5p	55	2.23
POLR2D	2	let-7b-5p, let-7c-5p	51	2.18
CASP3	2	let-7b-5p, let-7c-5p	56	2.05
NUP98	2	let-7b-5p, let-7c-5p	40	2.05
RB1	2	let-7b-5p, let-7c-5p	51	1.95
CDKN1A	2	let-7b-5p, let-7c-5p	68	1.88
CCND1	2	let-7b-5p, let-7c-5p	52	1.49
STAT3	2	let-7b-5p, let-7c-5p	51	1.39
MAP3K1	2	let-7b-5p, let-7c-5p	69	1.31
ITGB3	2	let-7b-5p, let-7c-5p	40	1.28
MAP3K7IP2	2	let-7b-5p, let-7c-5p	50	1.25

*Based only on miRSystem report for let-7b/c-5p, and miR-210-3p, -331–3p. O/E: ratio observed/expected.

Selection: O/E >= 1.0, Hits > 1, Pathways >= 1; if Hits = 2, then Pathways >= 40 or O/E > 10.

We further explored the functional interactions between common targets of DE miRNAs, as well as their functional implications, by constructing a PPI network and performing pathway analyses on it. This allowed us to identify not only functional types of interaction (i.e. activation/catalysis, inhibition, complex formation/binding, or predicted interaction), but also functionally related target clusters (network modules), as well as proteins likely linking different biological processes/pathways (network hubs, i.e. proteins showing the larger numbers of interactions within the network). The resulting full network consisted of 227 nodes and was enriched for pathways highly related to responses to growth factors, adhesion molecules and immunological stimulation, as well as morpho-/organogenesis, including the PI3K-Akt, MAPK, FoxO, Ras, IL4-mediated, TGF-β, integrin, Jak-STAT and p53 signaling pathways, focal adhesion and ECM-receptor interactions, among the most significantly enriched terms, and cell cycle regulation by BTG proteins, signaling by activin, Fas, SMAD2/3, EPHA, growth hormone and TNF receptors, regulation of hypoxia and oxygen homeostasis by HIF-1-α, and oxidative stress response, among the pathways with the higher proportions of proteins from the network in the pathway (Supplementary Table 4, Module: All).

**Figure 3:**
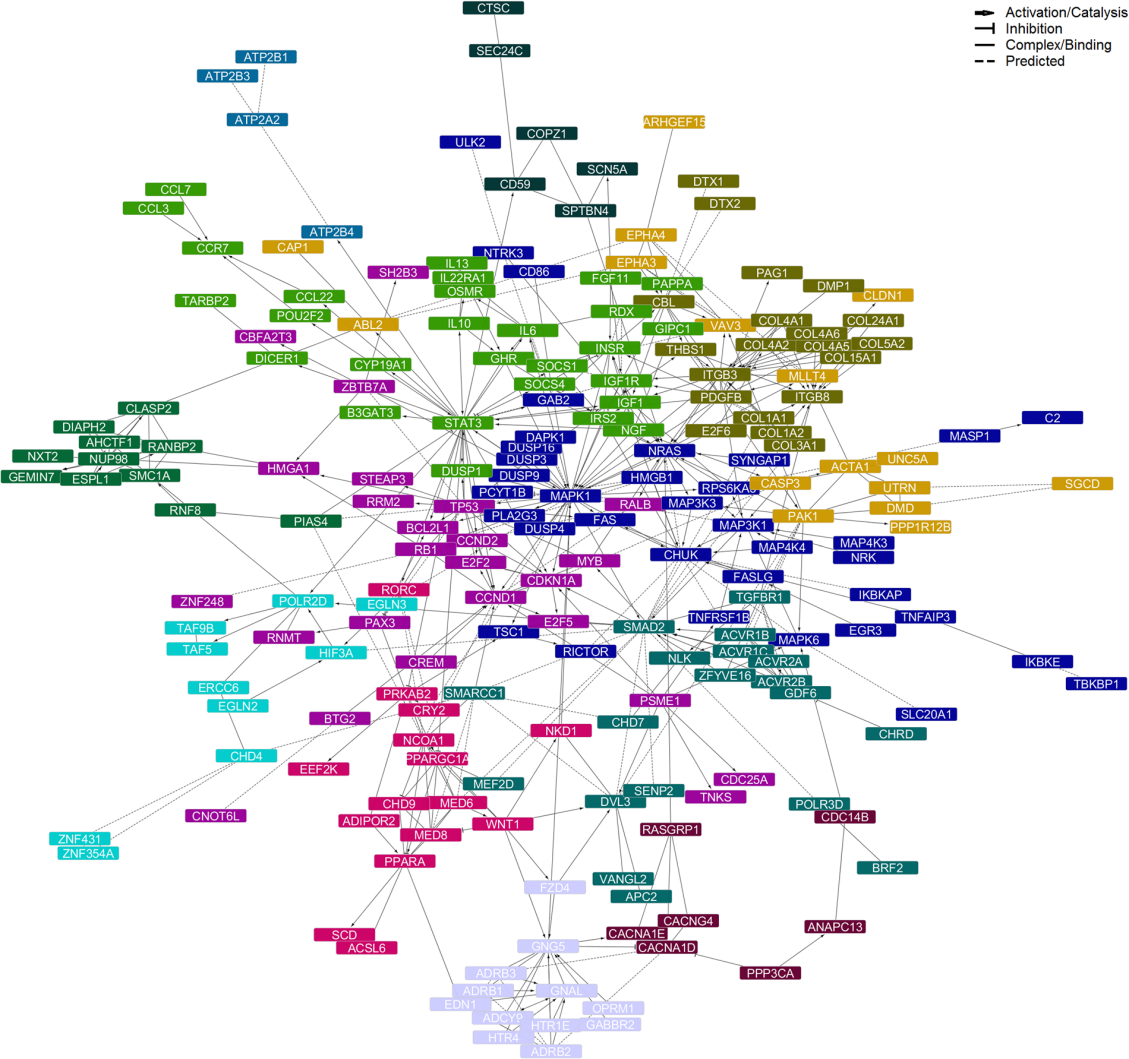
Protein-protein interaction network generated for shared miRNA target genes. After integration of miRSystem and miRTargetLink target gene lists, those genes predicted to be targeted by more than one of the differentially expressed miRNAs served as input for the network creation using the Reactome FI app for Cytoscape. Disconnected nodes and isolated clusters of less than 4 proteins were excluded from the network view and module enrichments. Information on paired protein interactions is available in the Supplementary Table 3. Top results from the pathway enrichment analyses for the network and network modules can be found in Table 3, and Supplementary Table 4.

The main network (Fig. 3, Supplementary Table 5) was conformed by 208 nodes, 480 edges (interactions), and 13 modules (clusters). As network hubs, we identified MAPK1 (38 interactions), STAT3 (31 interactions), ITGB3 and SMAD2 (22 interactions). In a lesser extent, NRAS and TP53 (18 interactions), as well as CHUK and PDGFB (16 interactions), were also highlighted. With the exception of MAPK1, which was a predicted target of miR-1307-3p and miR-185-3p, these network hub molecules were predicted targets for let-7b/c-5p miRNAs (Table 3). Pathway enrichment analysis of the network modules revealed that the largest clusters of proteins are involved in the responses to immune stimulation and growth signals, cell cycle regulation and tissue/organ morphogenesis, while smaller clusters play roles in hypoxia, oxytocin signaling, platelet homeostasis and cardiac conduction (Table 4, Supplementary Table 4). All these modules are closely linked through the aforementioned hub proteins. To illustrate this functional connectivity further, we mapped those miRNA targets observed within the Pathways in Cancer KEGG diagram as, due to the integrative nature of this pathway and its relation to cellular proliferation, growth and differentiation, we observed the highest proportion of proteins from the network in this term (Supplementary Fig. 1). In summary, the results from our analysis support the involvement of the TGF-β family of growth and differentiation factors, inflammation and semaphorin signaling, which may impair organ fetal organogenesis and postnatal lung and cardiac functions, in the severe pathophysiology of CDH with poor outcomes.

**Table 4:** Summary of main findings from the pathway analysis (by module) of the network created for shared target genes between top differentially expressed miRNAs in the Death/CLD group of CDH children.

Nodes	Top Pathways	Proteins
227	Signaling pathways activated in response to growth factors and adhesion molecules; tissue and organ morphogenesis.	
36	TNF receptor and MAPK signaling pathways; signaling by NGF; innate immune response; response to hormones and growth factors	C2, CD86, CHUK, DAPK1, DUSP16, DUSP3, DUSP4, DUSP9, EGR3, FAS, FASLG, GAB2, HMGB1, IKBKAP, IKBKE, MAP3K1, MAP3K3, MAP4K3, MAP4K4, MAPK1, MAPK6, MASP1, NRAS, NRK, NTRK3, PCYT1B, PLA2G3, RICTOR, RPS6KA3, SLC20A1, SYNGAP1, TBKBP1, TNFAIP3, TNFRSF1B, TSC1, ULK2
28	Jak-STAT, FoxO and PI3K-Akt signaling pathways; inflammation	B3GAT3, CCL22, CCL3, CCL7, CCR7, CYP19A1, DICER1, DUSP1, FGF11, GHR, GIPC1, IGF1, IGF1R, IL10, IL13, IL22RA1, IL6, INSR, IRS2, NGF, OSMR, PAPPA, POU2F2, RDX, SOCS1, SOCS4, STAT3, TARBP2
25	Cell cycle regulation and p53 signaling	BCL2L1, BTG2, CBFA2T3, CCND1, CCND2, CDC25A, CDKN1A, CNOT6L, CREM, E2F2, E2F5, HMGA1, MYB, PAX3, PSME1, RALB, RB1, RNMT, RRM2, SH2B3, STEAP3, TNKS, TP53, ZBTB7A, ZNF248
20	ECM organizaion, focal adhesion, integrin signaling; platelets	CBL, COL15A1, COL1A1, COL1A2, COL24A1, COL3A1, COL4A1, COL4A2, COL4A5, COL4A6, COL5A2, DMP1, DTX1, DTX2, E2F6, ITGB3, ITGB8, PAG1, PDGFB, THBS1
19	TGF-β, Wnt and Hippo signaling pathways	ACVR1B, ACVR1C, ACVR2A, ACVR2B, APC2, BRF2, CHD7, CHRD, DVL3, GDF6, MEF2D, NLK, POLR3D, SENP2, SMAD2, SMARCC1, TGFBR1, VANGL2, ZFYVE16
16	EPH-Ephrin signaling, axon guidance; cell migration	ABL2, ACTA1, ARHGEF15, CAP1, CASP3, CLDN1, DMD, EPHA3, EPHA4, MLLT4, PAK1, PPP1R12B, SGCD, UNC5A, UTRN, VAV3
15	Lipid and energy metabolism	ACSL6, ADIPOR2, CHD9, CRY2, EEF2K, MED6, MED8, NCOA1, NKD1, PPARA, PPARGC1A, PRKAB2, RORC, SCD, WNT1
12	Intracellular signaling	ADCY9, ADRB1, ADRB2, ADRB3, EDN1, FZD4, GABBR2, GNAL, GNG5, HTR1E, HTR4, OPRM1
11	Mitosis	AHCTF1, CLASP2, DIAPH2, ESPL1, GEMIN7, NUP98, NXT2, PIAS4, RANBP2, RNF8, SMC1A
10	Hypoxia; oxygen homeostasis	CHD4, EGLN2, EGLN3, ERCC6, HIF3A, POLR2D, TAF5, TAF9B, ZNF354A, ZNF431
7	MAPK, oxytocin, calcium signaling	ANAPC13, CACNA1D, CACNA1E, CACNG4, CDC14B, PPP3CA, RASGRP1
6	Protein transport and modification	CD59, COPZ1, CTSC, SCN5A, SEC. 24 C, SPTBN4
4	Platelet homeostasis; cardiac conduction	ATP2A2, ATP2B1, ATP2B3, ATP2B4

### Circulating DE miRNAs as potential prognostic biomarkers and/or therapeutic targets for CLD in CHD children

**Figure 4:**
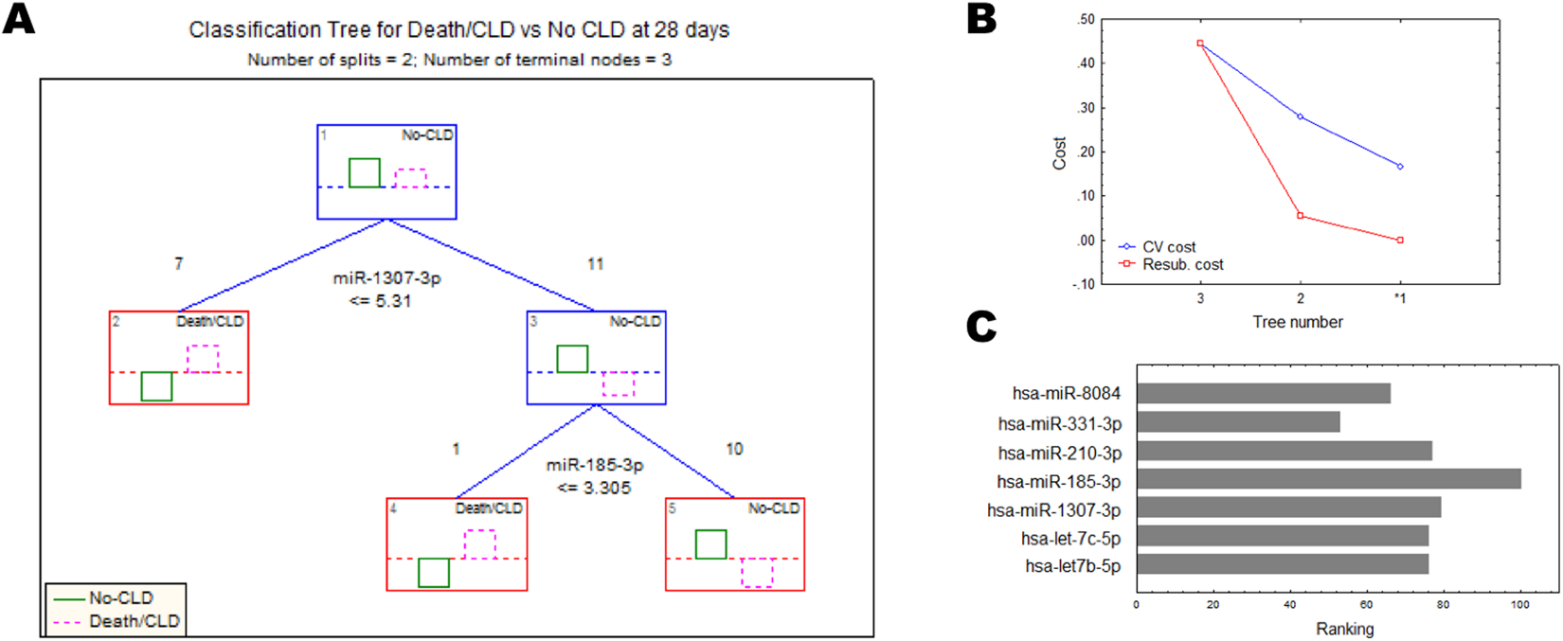
Classification of cases was used to obtain the relative importance of differentially expressed circulating miRNAs for the Death/CLD condition outcome. The corresponding chosen classification tree **(A)**, the costs of the cross-validation procedure **(B)** and the predictor importance ranking **(C)** for the chosen tree (number 1, *) are shown.

Finally, because of the high overlapping and redundant nature of miRNA target genes and pathways, we wished to investigate the relative importance of dysregulations at 24 h of the circulating DE miRNAs for the outcomes of Death/CLD at 28 days after birth in CDH children, in order to rank our DE miRNAs for future studies on their potential as prognostic biomarkers for CLD in CDH, and/or therapeutic targets. For this, we used C&RTs and found that most Death/CLD cases (7/8) could be differentiated from those No-CLD cases through the expression values of miR-1307-3p (importance rank: 79, split constant: 5.31), while miR-185-3p was important to separate the remaining set of Death/CLD cases initially classified as No-CLD within the first split (importance rank: 100, split constant: 3.305) to achieve a complete (100%) classification accuracy (Fig. 4A), using the lower cost (cross-validation cost: 0.167, resubstitution cost: 0), most suitable tree (Fig. 4B). From this algorithm, we obtained then a relative higher ranking of miR-185-3p and miR-1307-3p, closely followed by miR-210-3p (importance rank: 77), and the let-7b/c-5p miRNAs (importance rank: 76) (Fig. 4C), even though these latter were not included in the split criteria.

## Discussion

In this study, we identified seven miRNAs differentially expressed at 24 h of life in the blood of CDH newborns who developed CLD or died within 28 days after birth, and showed that these not only correlate with different measures of pulmonary dysfunction, but also interconnect to participate in processes crucial to establish proper morphology and function of the heart and lungs. Our results suggest that a subset of these miRNAs might hold the potential to serve as prognostic circulating biomarkers for the development of CLD in CDH newborns, which should be properly evaluated in an extended cohort, and implicate TGF-β and semaphorin signaling, as well as inflammatory responses, in the development of CLD in CDH.

The rate of mortality and disability in the long-term among CDH patients depends, in great extent, on the presence and severity of PH^19^. Persistent problems in surviving children, such as long-term oxygen dependency, need for mechanical ventilation, persistent wheezing, and increased risk for pulmonary infections^20–22^, suggest that a combination of mechanical ventilation-induced damage and primary defects of the lung and pulmonary vascular structures are responsible for the cardiopulmonary abnormalities observed in CDH children^2^. The contributions of miRNAs to lung morphogenesis during fetal development, and to inflammatory processes during pulmonary disease, have been attractive topics for research in past years. This led to the identification of a number of these molecules, including miR-210 and let-7 family members, as important regulators of normal and abnormal lung development and function^23^, and of pulmonary arterial remodeling in response to vessel injury and cellular stressors, such as hypoxia^24^. Consistently, members of the let-7 family of miRNAs and miR-1307 have been implicated in the severity of PH in systemic scleroderma^25^, miR-185 has been shown to contribute to the oxidative stress-mediated, hyperoxia-induced death of lung epithelial cells during acute lung injury and acute respiratory distress syndrome^26^ and, together with miR-210, contribute to the rapid progression of pulmonary fibrosis^27^. Further, miR-210, together with miR-331, has been associated with cardiovascular disease and markers of systemic inflammation in human immunodeficiency virus (HIV)-1 infection^28^.

The hypoxia-induced miR-210 has been previously implicated in PH, other lung diseases and ischemic heart disease^29–32^. We suggest that this miRNA also contributes to the development of CLD and the severity of PH as outcomes in CDH newborns. Interestingly, though, miR-210 presented a lower importance than miR-185 and miR-1307 to differentiate CDH cases within the Death/CLD group from those in the No-CLD group. We can speculate that these latter miRNAs regulate distinctive pathways that contribute to progression of CLD in a greater extent than the former, which might be implicated in pathways contributing to PH pathogenesis. Similarly, the previously reported PH-associated miR-21, miR-204, and clusters miR-143/145, miR-17-92 and miR-130/301, as well as the CDH-associated miR-200b and miR-10a might not have shown significance in our study because of a reduced relevance for severity and progression of CLD in CDH newborns, compared to the pathogenesis of each individual disease. Moreover, most observations on miRNA dysregulations in CDH and PH come from studies in animal or cellular models, which may not fully translate to the human pathobiology.

It is well-known that the TGF-β superfamily plays crucial roles in pre- and post-natal lung development, importantly shaping alveolarization and controlling the extracellular matrix composition and tissue homeostasis, among other functions. Not surprisingly, its involvement in pulmonary and cardiovascular diseases has been largely described^33–36^. Because the TGF-β canonical and non-canonical signaling pathways overlap with a number of pathway terms highly enriched in our PPI network^34,37^, our results provide evidence for the involvement of the TGF-β superfamily in the development of CLD in CDH newborns, providing support to the hypothesis that a primary disruption in lung vasculature and airway development accompany the diaphragmatic defect in CDH, and that the extent of the resulting cardiopulmonary dysfunction contributes to the development of CLD in CDH newborns. Further support to this hypothesis is found through our observation that major genes participating in the semaphorin signaling, involved in development and regulation of immune responses through guidance cues^38,39^, are predicted targets of the miRNAs we found dysregulated in blood of CDH children with severe outcomes.

The O/E ratio of fetal lung volume measured by ultrasound or magnetic resonance imaging (MRI) has been proposed to serve as prognostic biomarker for mortality, the need for ECMO and development of CLD^40,41^. Our study is, to our knowledge, the first to assess the differential miRNA blood profiles related to death and the development of CLD in CDH, and provide an extensive analysis of functional implications and hierarchies of miRNA dysregulations which not only suggests potential for measurements of circulating miRNAs as prognostic biomarkers, but also uncovers crucial regulators with potential for therapeutic purposes. It has already been demonstrated that less severe lung hypoplasia in CDH is associated with compensatory upregulation of miR-200b, and that treatment with this miRNA at an early developmental stage improves lung growth in the nitrofen-induced CDH rodent model^42^.

We acknowledge the small sample size of our study is an important limitation, providing small power for statistical testing. Our miRNA candidates would require validation in a larger, well-characterized cohort. Another limitation in our study is the lack of repeated measures in a time series, which would have allowed us to identify changes induced by ECMO and other external factors that influence the development of CLD in CDH newborns. Similarly, the unavailability of tissue-specific miRNA expression limits our ability to interpret the biological meaning of our pathway and network analyses. However, all of these pathways have been described in the context of lung development, PH, and inflammation, all being a part of the pathophysiology of CDH. Considering array measures of small non-coding RNAs may be biased only to those miRNAs and other small RNAs that are part of the array, it is possible that using RNA-seq would have led to discovery of additional, novel circulating miRNAs associated with severe outcomes in CDH. This is an important avenue of future research. Finally, we also acknowledge the potential overfitting of our C&RTs and, although we obtained similar results using two different algorithms (data not shown) for this same reason, the aim of this analysis was not to provide proof of the biomarker performance of our identified miRNAs, but to help us in ranking them for future follow-up studies.

In conclusion, we identified several miRNAs differentially expressed at 24 h of life in the blood of CDH newborns who developed CLD or died within 28 days after birth. Whether these miRNAs might serve as prognostic circulating biomarkers for the development of CLD in CDH newborns, and might be useful therapeutic targets, needs to be investigated in a larger cohort. Despite the aforementioned limitations, we believe our study provides encouraging evidence to ensure further validation of our present findings.

## Methods

### Sample cohort

Eighteen CDH patients with PH admitted to our neonatal intensive care unit (NICU) were prospectively enrolled in this study. Written informed consent was obtained from parents or legal representatives. The study was approved by the Institutional Review Board of the University Hospital Bonn and performed in accordance with the ethical standards described in the Declaration of Helsinki. Patients with severe concomitant malformations or insufficient echocardiographic data were excluded from the study.

### Standard CDH treatment protocol

Infants were intubated after delivery. Mechanical ventilation was started using gentle ventilation and permissive hypercapnia (if tolerated by the patient). The initial inspired oxygen fraction (FiO_2_) was 1.0, and infants received inhaled nitric oxide (iNO) therapy during the initial period of stabilization (iNO 20 ppm). FiO_2_ was titrated to achieve an arterial postductal partial oxygen pressure (PaO_2_) of 80–150 mmHg, and ventilator settings were adjusted to reach a partial pressure of carbon dioxide (PCO_2_) of 45–60 mmHg. Dobutamine and milrinone were administered to treat cardiac dysfunction, and norepinephrine and vasopressin were added to achieve a mean arterial blood pressure ≥40 mmHg. Infants were sedated with fentanyl and midazolam during the first days of life. Criteria for ECMO followed those published in the guidelines of the CDH Euro Consortium^13^: preductal oxygen saturation <85% or postductal saturation <70%, oxygenation index (OI) ≥40 and consistently present, increased PaCO_2_ > 70 mmHg with a pH < 7.15, a peak inspiratory pressure ≥28 cm H_2_O or mean airway pressure ≥17 cm H_2_O, or persistent systemic hypotension (mean arterial pressure <40 mmHg).

### Assessment of PH severity

Echocardiography was performed using a Philips CX50 CompactExtreme Ultrasound System with a S12-4 sector array transducer (Philips Healthcare, Best, The Netherlands). The pulmonary arterial pressure (PAP) was graded as <2/3 systemic pressure (mild PH), 2/3 systemic pressure to systemic pressure (moderate PH) or suprasystemic pressure (severe PH), as described by Keller *et al*.^14^. Assessment of PH included: (1) Ductus Arteriosus flow pattern; (2) intraventricular septum position; and (3) calculation of right ventricular systolic pressure from the tricuspid regurgitation (TR) jet, with an estimation of 5 mmHg for right atrial pressure. The angle of insonation was kept below 20°.

### Outcome data

Study personnel prospectively documented information on vital signs, blood gases and treatment data. The OI was calculated using the formula: OI = (MAP × FiO_2_ × 100)/PaO_2_, where MAP represents the mean airway pressure in cm H_2_O. For group allocation according to disease severity, a combined primary clinical endpoint was used, consisting of death or development of CLD within the first 28 days after birth (Death/CLD), or survival without CLD at 28 days of life (No-CLD). CLD was defined as oxygen dependency at 28 days of life according to the definition of bronchopulmonary dysplasia of Jobe and Bancalari^15^.

### RNA extraction and miRNA array hybridization

Post-pulmonary arterial blood samples were collected from 18 CDH newborns 24 hours after birth, via an indwelling arterial catheter in the femoral artery and using the PAXgene Blood RNA System (QIAGEN, Germany). Total RNA was isolated using the chemagic RNA Blood Kit special (CMG-1083, PerkinElmer, Baesweiler, Germany). The quantity and quality of the isolated RNA were assessed using an ND-1000 spectrophotometer (Peqlab Biotechnologie, Erlangen, Germany) and a BioAnalyzer 2100 (Agilent Technologies, Waldbronn, Germany), respectively. All samples had a concentration ≥20 ng/µl and an RNA integrity number (RIN) ≥6.9. Profiling of miRNAs was carried out from 250 ng of total RNA hybridized to Affymetrix GeneChip miRNA 4.0 arrays (Affymetrix, Santa Clara, CA), following manufacturer’s instructions. Poly(A) tailing and biotinylation were performed using the Affymetrix GeneChip Hybridization, Wash, and Stain Kit.

### Data analysis

Affymetrix CEL files were pre-processed following the robust multi-array analysis (RMA) and detection above background (DABG) workflow in the Affymetrix Expression Console (Affymetrix Santa Clara, CA) software. miRNA expression data was used as background-substracted, quantile-normalized and log2-transformed values. Individual probesets were considered present in a given sample if detection p < 0.05. After annotation, all probesets corresponding to RNAs other than mature miRNAs were removed. A miRNA was considered expressed in the dataset if the corresponding probeset was present in at least 12 of the 13 samples from newborns with 2/3 of systemic pressure. All expressed probesets were present in at least 16 of the 18 total samples.

### Statistical analysis

Statistical comparisons of demographic/clinical data and circulating miRNA expression levels between children in the Death/CLD and No-CLD groups was performed by means of two tailed Mann-Whitney U-tests, and a moderated t-test adjusted for multiple comparisons through the Benjamini-Hochberg (BH) method, respectively. Statistical significance was set to raw p < 0.05 values for demographic/clinical data and “significantly changed” miRNAs, whereas “differentially expressed” (DE) miRNAs were considered those with BH-adjusted p < 0.05 and expression change ≥1.0. Correlation coefficients between clinical parameters and expression levels of DE miRNAs were obtained through the Pearson’s method.

### Target genes

DE miRNAs were submitted for “miRNAs to Target Genes” analysis on miRSystem^16^, using default settings and the expression changes between Death/CLD and No-CLD groups as weights. DE miRNAs not found in the miRSystem database were queried individually on miRTargetLink^17^ to obtain the evidence-based target genes. Overlaps between individual target lists were then identified. Furthermore, it was investigated whether the significantly changed miRNAs are expressed in lung tissue and/or there is evidence that they have targets in lung, by searching each miRNA in the interactive multi-omics-tissue atlas (IMOTA)^18^.

### Protein-protein interactions and biological pathways

To investigate the functional implications of miRNA dysregulations in severe CDH outcomes, we created a protein-protein interaction (PPI) network of molecules targeted by at least two of our DE miRNAs with pathway annotations in miRSystem using the Reactome FI app for Cytoscape 3.5.1. We clustered the network and fetched the annotations to show the types of functional relationships. All unconnected nodes, as well as clusters conformed by less than four proteins, were removed from the network view. The full network and network modules (clusters) not hidden from view were analyzed for enriched biological pathways. Enrichment results were filtered to keep only those terms with false discovery rate (FDR) <0.01 and a number of module genes in the term > 2.

### Relative importance of DE miRNAs

To investigate the relative importance of DE miRNAs for condition outcome, we employed a classification and regression tree (C&RT)-style algorithm to apply an exhaustive search for univariate splits for Death/CLD or No-CLD class prediction. The tree was generated using STATISTICA 7 (StatSoft), using all seven DE miRNAs as continuous predictor variables and the following settings: chi-square goodness of fit, estimated prior probabilities, pruning on misclassification error, and a seed for random number generator = 10, plus 3-fold cross-validation.

## Electronic supplementary material

Supplementary Dataset 6

Supplementary Dataset 1

Supplementary Dataset 2

Supplementary Dataset 3

Supplementary Dataset 4

Supplementary Dataset 5
